# Perfusion deficits in thrombolysis-treated acute ischemic stroke patients with negative or positive diffusion-weighted imaging

**DOI:** 10.1186/s12883-023-03427-9

**Published:** 2023-10-21

**Authors:** Cuiting Zhu, Wei Qin, Jihua Xu, Wenli Hu

**Affiliations:** Department of Neurology, Beijing Chaoyang Hospital, Capital Medical University, 8 Gongti South Road, Chaoyang District, Beijing, 100020 P.R. China

**Keywords:** Intravenous tissue-type plasminogen activator, CT perfusion, DWI, Perfusion deficits, Ischemic stroke

## Abstract

**Objective:**

Magnetic resonance imaging (MRI) and CT perfusion may provide diagnostic information for intravenous tissue-type plasminogen activator (IV t-PA) administration in acute ischemic stroke (AIS) patients. We aimed to compare the clinical features and perfusion deficits of diffusion weighted imaging (DWI)-negative and DWI-positive AIS patients.

**Methods:**

This retrospective and observational study included thrombolysis-treated AIS patients undergoing multimodel CT imaging before treatment and DWI after treatment between 2021 and 2022. Two experienced neuroradiologists blindly and independently examined the images to identify perfusion deficits in AIS patients. The patients were divided into DWI-positive and DWI-negative groups based on visible hyperintense lesions on DWI. A modified Rankin scale (mRS) score of ≤ 2 indicated good functional outcomes at discharge. Sensitivity analysis was conducted to determine whether CT perfusion was an independent predictor of positive DWI imaging on follow-up.

**Results:**

This study included 151 patients, of whom 35 (23.2%) patients were DWI-negative on follow-up. These DWI-negative patients were less likely to have a medical history of atrial fibrillation; they had lower triglyceride levels, a shorter admission time, lower National Institutes of Health Stroke Scale (NIHSS) scores after IV t-PA and lower mRS scores at discharge, and had better functional outcomes. A total of 37.1% of DWI-positive and 25.7% of DWI-negative patients had vascular stenosis (*P* = 0.215). A total of 47.4% of DWI-positive and 37.1% of DWI-negative patients had CT perfusion deficits (*P* = 0.284). A total of 73.5% of patients with normal CT perfusion had positive DWI, while 19.1% of patients with perfusion deficits had negative DWI. The sensitivity and specificity of NCCT were 14.8% and 97.1% (Kappa = 0.061, *P* = 0.074), CTP was 47.4% and 62.9% for predicting DWI lesion (Kappa = 0.069, *P* = 0.284).

**Conclusions:**

About 23.2% of AIS patients who received intravenous thrombolysis treatment did not have a relevant DWI-MRI lesion on follow-up. Over one-third of patients in the DWI-MRI negative group showed CT perfusion deficits, with a sensitivity of 47.4% for predicting DWI lesions in non-mechanical thrombectomy patients.

## Introduction

Intravenous tissue-type plasminogen activator (IV t-PA) was administered to acute ischemic stroke (AIS) patients after screening with a head CT to exclude intracerebral hemorrhage. Magnetic resonance imaging (MRI) with diffusion weighted imaging (DWI) is often used to diagnose stroke and guide treatment decisions. However, it is time-consuming and has several limitations. Moreover, previous studies discovered that patients with persistent neurological deficits had false negative DWI. It was reported that 6.8 29% of patients were DWI-negative [1–4]. DWI fails to detect AIS in a substantial minority of patients due to underestimated hyperacute ischemia [1]. Several randomized clinical trials have demonstrated that endovascular stroke therapy for acute stroke has significantly improved [5, 6]. More patients treated with IV t-PA at a later time window benefit from CT perfusion (CTP) imaging-based definitions of eligibility [7]. CT angiography (CTA) and CTP can detect intracranial vascular stenosis or occlusion and perfusion deficits in patients with different types of ischemic stroke. The sensitivity of CTP varies significantly due to the heterogeneity in patient characteristics, CTP spatial and temporal resolution, and postprocessing methods [8]. CT imaging is superior to MRI in terms of acquisition speed, absence of screening questionnaires, scanner availability, cost, and processing time [9]. Evaluating the infarct core volume using CTP could provide a timely assessment of tissue perfusion status in hyperacute stroke and the long-term clinical prognosis of AIS patients. We aimed to determine whether CT imaging provides further diagnostic information for the evaluation of DWI-negative patients with ischemic stroke.

This study aimed to compare the clinical features and CT perfusion deficits of DWI-negative AIS patients to DWI-positive AIS patients. The clinical value of CT perfusion in identifying patients with non-mechanical thrombectomy treatment was also investigated to help decide whether to adopt intravenous thrombolysis in AIS patients.

## Method

### Study design and patients

**Fig. 1:**
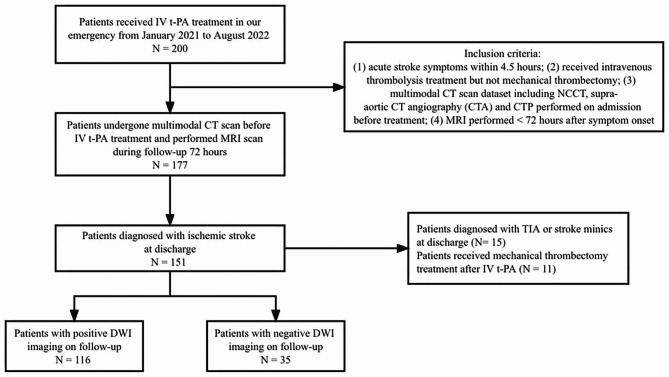
Study flow chart

The design of this study was approved by the Ethics Committee of Beijing Chaoyang Hospital, Capital Medical University, and was performed in accordance with the declaration of Helsinki. A retrospective review of consecutive patients admitted to the stroke unit of our hospital who presented with AIS or transient ischemic attack (TIA) and received IV t-PA treatment between January 2021 and August 2022 was performed. AIS was diagnosed using WHO criteria (acute episode of neurological deficit, lasting less than 24 h in the case of TIA, more than 24 h in the case of an ischemic stroke) [10]. The subtype of ischemic stroke was diagnosed by an experienced neurologist according to the established criteria of TOAST [11]. The inclusion criteria for this study were: (1) acute stroke symptoms within 4.5 h; (2) received intravenous thrombolysis treatment but not mechanical thrombectomy; (3) multimodal CT examination dataset including non-contrast CT brain (NCCT), supra-aortic CTA and CTP performed on admission before treatment; (4) MRI performed < 72 h after symptom onset. Exclusion criteria were: (1) Patients diagnosed as TIA or stroke mimics; (2) Patients who had incomplete clinical data. (Fig. 1).

### Demographic data

Data were recorded on demographic characteristics and vascular risk factors that included age, sex, systolic and diastolic blood pressure (BP) at presentation, body mass index, duration of admission in days, history of hypertension, diabetes mellitus, hyperlipidemia, atrial fibrillation, ischemic stroke, and coronary artery disease, current smoking and drinking status and time of symptom onset. Neurological deficit symptoms were recorded by admission and after treatment National Institutes of Health Stroke Scale (NIHSS) score [12], Modified Rankin Scale (mRS) score [13] at discharge. The mRS scores of 3–6 were used to define poor functional outcomes at discharge. Laboratory data included cholesterol, triglycerides, HDL-C, LDL-C, Lipoprotein (a), creatinine, HbA1c, Homocysteine (Hcy), and uric acid).

### CTP and DWI acquisition and postprocessing

Multimodal CT examinations including NCCT, CTP, and CTA were performed on a 64-section multidetector scanner (Revolution Frontier, GE Healthcare, Siemens) [14]. Standard NCCT was acquired (5 mm slices) followed by CTP using a SOMATOM Emotion 16 (Siemens GE Healthcare). Perfusion maps including cerebral blood flow (CBF), cerebral blood volume (CBV), time to peak (TTP), and mean transit time (MTT) were produced by using the fast-processing of ischemic stroke (F-STROKE) software [15].

MRI examinations including conventional T1-weighted, T2-weighted, fluid-attenuated inversion recovery imaging, susceptibility weighted imaging (SWI), and DWI were performed on a 3.0-T MRI scanner (Prisma; Siemens AG, Erlangen, Germany) with a 32-channel array head coil. We rated the total MRI burden of cerebral small vessel disease (CSVD) on an ordinal scale from 0 to 4 [16], by counting the presence of each of the 4 MRI features of CSVD presence of lacunes and cerebral microbleeds (CMBs) were defined as the presence of one or more lacunes (1 point if present) or any CMB (1 point if present). Presence of perivascular spaces (PVS) was counted if there were moderate to severe (grade 2–4) PVS in the basal ganglia (1 point if present). Presence of white matter hyperintensities (WMH) was defined as either (early) confluent deep WMH (Fazekas score 2 or 3) or irregular periventricular WMH extending into the deep white matter (Fazekas score 3) (1 point if present).

### Image review and interpretation

The imaging data included DWI, NCCT, CTA, and CTP (CBV, CBF, MTT, and TTP) images was performed blindly and independently by two experienced neuroradiologists. Ischemic core volume was defined as relative CBF < 30% and penumbra volume as T-max > 6s in normal tissue [17]. Perfusion deficits were defined as regions with abnormality of CTP map, decreased of CBF or CBV, or/and increased T-max or MTT compared with the contra-lateral side [14]. Using visible hyperintense lesion on DWI as the criterion standard, patients were divided into DWI-positive and DWI-negative groups. DWI-negative patients were defined as persistent focal neurological dysfunction for more than 24 h but no areas of hyperintensity observed on DWI. Fluid-attenuated inversion recovery (FLAIR) images were reviewed to ensure that the perfusion deficits did not correspond to a region of old infarction. Supra-aortic CT angiography (CTA) was evaluated for the presence of underlying vascular stenosis that corresponded to the regional perfusion deficits. Vascular stenosis was presented as > 50% stenosis of intracranial and extracranial large vessels of CTA. In cases of discrepancies, a consensus read was performed with both readers and a senior and more experienced reviewer who was not involved in the initial grading. Inter-observer agreement on quantitative perfusion parameters were assessed in terms of the intraclass correlation coefficient (95% confidence interval), which was 0.80.

### Statistical analysis

Categorical variables were presented as percentages and continuous variables as mean with standard deviation or median with interquartile range. For categorial variables, chi-square or Fisher’s exact test was used to compare baseline characteristics between negative and positive DWI lesion test results. The t-test or Mann-Whitney U test was performed for continuous variables. The sensitivity and specificity of CTP results for predicting positive DWI lesions were determined using Paired chi-square test and Kappa consistency checking. Statistical analyses were performed using SPSS 26.0. *P* < 0.05 was considered statistically significant.

## Results

### Demographic profile

In the present study, 200 patients with acute stroke symptoms completed multimodal CT examination within 4.5 h of symptom onset, and MRI examination within 72 h of symptom onset from Jan 2021 to Aug 2022. A total of 151 patients with the final diagnosis of stroke were included in this study after excluding patients with TIA, stroke mimics, and other causes. The mean age was 64.8 ± 11.4 years, and 102 (67.5%) patients were male. There were 35 (23.2%) patients with negative DWI on follow-up. DWI-negative patients were less likely to have a history of atrial fibrillation, had lower triglyceride levels, had shorter admission duration, had a lower NIHSS score after IV t-PA and mRS score at discharge, and better function outcomes compared to DWI-positive patients (Table 1). There was no significant difference in age and gender between the negative and positive DWI groups.

**Table 1:** Demographic and baseline characteristics

Characteristics	DWI-negative (n = 35)	DWI-positive (n = 116)	*P* value
Age, years, mean ± SD	64.7 ± 8.8	64.8 ± 12.2	0.971
Sex, males, n (%)	22.0(62.9)	80.0(69.0)	0.499
NIHSS score before IVT	3.0(1.5-4.0)	4.0(2.0–8.0)	0.100
NIHSS score after IVT	1.0(0–2.0)	2.0(0–5.0)	0.045
Vessel stenosis or occlusion, n (%)	9(25.7)	43(37.1)	0.215
DNT, minute	57.0(51.0–66.0)	59.0(50.0–70.0)	0.793
HT, n (%)	26.0(74.3)	74.0 (63.8)	0.250
DM, n (%)	13.0 (37.1)	41.0 (35.3)	0.846
CHD, n (%)	8.0 (22.9)	20.0 (17.2)	0.454
AF, n (%)	0(0.0)	14.0 (12.1)	0.021
Hyperlipidemia, n (%)	16.0 (45.7)	45.0 (38.8)	0.465
CI history, n (%)	6.0 (17.1)	16.0 (13.8)	0.622
Smoking, n (%)	19.0 (54.3)	58.0 (50.0)	0.657
Drinking, n (%)	11.0 (31.4)	39.0 (33.6)	0.809
SBP, mmHg	154.0 (135.5-164.5)	154.0 (139.5–163.0)	0.650
DBP, mmHg	82.0(77.0–90.0)	82.5(75.5–94.0)	0.610
BMI	24.8(23.4–26.8)	25.0(23.0-27.7)	0.985
Blood glucose level, mmol/l	7.8(6.8–9.8)	7.0(5.7–9.9)	0.829
TC, mmol/L	4.8 ± 1.1	4.7 ± 1.1	0.949
TG, mmol/L	2.2(1.8–3.2)	3.1(2.4–3.9)	0.018
HDL-C, mmol/L	1.1(1.0-1.2)	1.1(0.9–1.2)	0.334
LDL-C, mmol/L	3.0 ± 1.1	3.1 ± 1.0	0.763
Lipoprotein (a), mg/dL	16.3(9.2–20.3)	19.1(9.8–38.5)	0.341
Cr, umol/L	73.8 ± 15.4	68.7 ± 15.6	0.092
HbA1c, %	6.4(5.8-7.0)	6.0(5.7–7.2)	0.863
Hcy, umol/L	12.0(10.0–15.0)	12.0(11.0–16.0)	0.315
Uric acid, umol/L	344.0 ± 102.0	337.7 ± 95.5	0.739
Duration of admission in days	9.0(8.0–10.0)	10.5(9.0–12.0)	< 0.001
mRS at discharge	0(0–1.0)	2.0(1.0–3.0)	< 0.001
Poor function outcome	1(2.9)	37(31.9)	< 0.001

Abbreviations: DWI diffusion weighting imaging, SD standard deviation, NIHSS National Institutes of Health Stroke Scale, IVT Intravenous thrombolysis, DNT door to needle time, HT hypertension, DM diabetes, CHD coronary heart disease, AF atrial fibrillation, CI cerebral infarction, SBP systolic blood pressure, DBP diastolic pressure, BMI body mass index, TC total cholesterol, TG triglyceride, Cr creatinine, Hcy homocysteine, mRS modified Rankin scale

### Clinical features of different CT perfusion results

A total of 68 (45.0%) patients had CT perfusion deficits with a median age of 62.0 (55.5. 70.5) years (Table 2). A total of 83 (55.0%) patients had normal CT perfusion with a median age of 65.0 (59.0 75.0). There were 51 (61.4%) patients in normal CT perfusion and 57 (83.8%) patients in the CT perfusion deficits group diagnosed with anterior circulation AIS. There were 61 (73.5%) DWI-positive patients in normal CT perfusion and 13 (19.1%) DWI-negative patients in CT perfusion deficits group. Patients with perfusion deficits were older, more likely to have history of diabetes and atrial fibrillation, have a longer hospital stay, and have poor function outcomes compared to patients with normal CT perfusion. There were statistically significant differences in age and gender between the two groups.

**Table 2:** Clinical features of different CT perfusion results

Characteristics	Normal CTP (n = 83)	Abnormal CTP (n = 68)	*P* value
Age, years, mean ± SD	62.0(55.5–70.5)	65.0(59.0–75.0)	0.043
Sex, males, n (%)	64(77.1)	38(55.9)	0.006
Negative DWI	22(26.5)	13(19.1)	0.284
NIHSS score before IVT	3.0(2.0–6.0)	4.0(2.0-8.5)	0.253
Anterior circulation, n (%)	51(61.4)	57(83.8)	0.002
Vessel stenosis or occlusion, n (%)	17(20.5)	35(51.5)	< 0.001
NCCT, n (%)	10(12.0)	8(11.9)	0.984
HT, n (%)	53(63.9)	47(69.1)	0.496
DM, n (%)	23(27.7)	31(45.6)	0.023
CHD, n (%)	13(15.7)	15(22.1)	0.314
AF, n (%)	2(2.4)	12(17.6)	0.001
Hyperlipidemia, n (%)	29(34.9)	32(47.1)	0.131
CI history, n (%)	11(13.3)	11(16.2)	0.612
Smoking, n (%)	48(57.8)	29(42.6)	0.063
Drinking, n (%)	31(37.3)	19(27.9)	0.222
LI, n (%)	43(51.8)	33(48.5)	0.689
WMH, n (%)	59(71.1)	46(67.6)	0.648
CMB, n (%)	10(12.0)	10(14.7)	0.632
PVC, n (%)	61(73.5)	42(61.8)	0.124
Duration of admission in days	9.5(8.0–12.0)	10.5(9.0–12.0)	0.037
mRS at discharge	1.0(0–2.0)	1.5(0-3.5)	0.069
Poor function outcome	13(15.7)	25(36.8)	0.003

Abbreviations: CTP CT perfusion, DWI diffusion weighting imaging, SD standard deviation, NIHSS National Institutes of Health Stroke Scale, IVT Intravenous thrombolysis, NCCT non-contrast CT brain, HT hypertension, DM diabetes, CHD coronary heart disease, AF atrial fibrillation, CI cerebral infarction, LI lacunar infarcts, WMH white matter hyperintensities, CMB cerebral microbleeds, PVC perivascular space, mRS modified Rankin scale

### Imaging results of CTP and CSVD score

Table 3 showed the results of CTP and CSVD score in patients with negative and positive DWI groups. There was no significant difference in vascular stenosis, CT perfusion deficits, and CSVD score between positive and negative DWI groups. It showed that 37.1% of DWI-positive patients and 25.7% of DWI-negative patients had vascular stenosis. There were 47.4% of DWI-positive patients and 37.1% of DWI-negative patients had CT perfusion deficits. The 73.7% of patients with ipsilateral stenosis had perfusion deficits. The sensitivity and specificity of CBV were 11.2% and 82.9% (Kappa= -0.030, *P* = 0.353), CBF 36.2% and 65.7% (Kappa = 0.012, *P* = 0.835), TTP 44.8% and 65.7% (Kappa = 0.069, *P* = 0.269), and MTT 44.8% and 65.7% (Kappa = 0.069, *P* = 0.269) for predicting DWI lesions. The sensitivity and specificity of NCCT were 14.8% and 97.1% for predicting DWI lesions (Kappa = 0.061, *P* = 0.074). The sensitivity and specificity of CTP were 47.4% and 62.9% for predicting DWI lesions (Kappa = 0.069, *P* = 0.284).

**Table 3:** The results of CTP and CSVD score in two group patients

Characteristics	DWI-negative (n = 35)	DWI-positive (n = 116)	*P* value
Anterior circulation, n (%)	25(71.4)	83(71.6)	0.989
Vascular stenosis or occlusion, n (%)	9(25.7)	43(37.1)	0.215
CT Perfusion deficits, n (%)	13(37.1)	55(47.4)	0.284
Decreased CBV, n (%)	6(17.1)	13(11.2)	0.353
Decreased CBF, n (%)	12(34.3)	42(36.2)	0.835
Delayed MTT, n (%)	12(34.3)	52(44.8)	0.269
Delayed TTP, n (%)	12(34.3)	52(44.8)	0.269
LI, n (%)	17(48.6)	59(50.9)	0.812
WMH, n (%)	22(62.9)	83(71.6)	0.327
CMB, n (%)	2(5.7)	18(15.5)	0.164
PVC, n (%)	26(74.3)	77(66.4)	0.379
CSVD score	1(0–2)	1(0–2)	0.411
0	18(51.4)	53(45.7)	
1	7(20.0)	24(20.7)	
2	7(20.0)	21(18.1)	
3	2(5.7)	5(4.3)	
4	1(2.9)	13(1.2)	

Abbreviations: CTP CT perfusion, DWI diffusion weighting imaging, CSVD cerebral small vessel disease, CBV cerebral blood volume, CBF cerebral blood flow, MTT mean transit time, TTP time to peak, LI lacunar infarcts, WMH white matter hyperintensities, CMB cerebral microbleeds, PVC perivascular spaces

## Discussion

In our retrospective study of 151 patients with AIS who were treated with intravenous thrombolysis, 23.2% of patients were DWI-negative on follow-up, of whom 37.1% had perfusion deficits on CTP imaging. Our results showed that DWI-positive patients had a longer admission duration, higher NIHSS scores after IV t-PA and mRS scores at discharge, and poor functional outcomes than DWI-negative patients. More than one-third of DWI-negative patients had perfusion deficits. Our study revealed a low concordance between the proportion of patients with perfusion deficits on CTP at admission and 72-hour DWI. The reason may be that CTP imaging failed to identify 38.6% patients with posterior circulation. Moreover, 19.1% of patients with lacunar infracts were difficult to detect using CTP imaging. Lacunar strokes and posterior circulation had smaller infarcts that could be missed in DWI imaging with large slice thickness [18]. Previous studies indicate that perfusion imaging may have prognostic value in anterior circulation acute ischemic stroke and aid in the selection of patients outside the time window for intravenous thrombolysis (IVT) or mechanical thrombectomy [19].

Our study revealed that 73.5% of patients had positive DWI in normal CT perfusion, while 19.1% of patients with perfusion deficits had negative DWI. CTP imaging revealed that 45% of AIS patients who received intravenous thrombolysis treatment had perfusion deficits. CTP imaging could help in the diagnosis of perfusion deficits in DWI-negative patients. In our study, 37.1% of ischemic stroke patients with negative DWI had perfusion deficits, involving anterior circulation in 84.6% and posterior circulation in 15.4% of patients. A previous study numerically and visually confirmed that CTP provides a stronger ischemic infarct core signal than NCCT [20]. CTP can select eligible AIS patients for reperfusion therapy based on the ischemic core and penumbra [21]. Whole-brain CT perfusion was used in a study to examine volumetric perfusion deficits in AIS patients, and discovered that perfusion deficits had the highest absolute volumes in MTT and T-max maps [22].

The DWI slice thickness and any slice gap must be reported to determine whether some DWI-negative cases were overlooked. In our study, 23.2% of ischemic stroke patients who received intravenous thrombolysis treatment were DWI-negative on follow-up, comparable to the previous 12.36 to 26% of patients treated with IV t-PA lacking imaging evidence of acute infarction on follow-up [23, 24]. A study on posterior circulation reported that 26% of acute lateral medullary infarction patients had negative DWI-MRI [25]. Our study discovered that DWI-negative patients had lower NIHSS scores after IV t-PA and mRS scores at discharge and better functional outcomes. These results were consistent with the observations of a nationwide prospective registry, the CNSR-III [26]. A previous study revealed that mild to moderate cerebral ischemia can cause isolated persistent synaptic failure without neuronal death [27]. This could explain why DWI-negative patients had lower NIHSS scores. A lower NIHSS score and small DWI lesion volume before IV t-PA was reported to be associated with a favorable outcomes [28, 29].

### Limitations

Our study had several limitations. The single-centre retrospective design and relatively small sample size could undermine the generalizability of our findings. Patients who underwent mechanical thrombectomy following intravenous thrombolysis or did not receive thrombolysis treatment were excluded. Our study showed no significant difference in CSVD scores between the two groups. Patients with large vessel occlusion and ischemic core volumes can be easily identified through CT imaging. More accurate imaging techniques and prevention follow-up for small lacunar infarcts need to be investigated. Further research is necessary to evaluate the significance of neurological examinations in a more comprehensive stroke population, including patients who received mechanical thrombectomy.

## Conclusion

On follow-up DWI-MRI, 23.2% of AIS patients who underwent intravenous thrombolysis had no relevant lesion. However, we discovered that over one-third of patients in the DWI-MRI negative group had CT perfusion deficits, with a sensitivity of 47.4% for predicting DWI lesions in non-mechanical thrombectomy patients. The use of CT perfusion evaluation in non-mechanical thrombectomy AIS patients before IV t-PA ensures acute treatment based on a valid diagnosis with a decreased risk of treating stroke mimics.

## Data Availability

The datasets used and/or analyzed during the current study available from the corresponding author on reasonable request.

## References

[1] Edlow BL, Hurwitz S, Edlow JA (2017). Diagnosis of DWI-negative acute ischemic stroke: a meta-analysis. Neurology.

[2] Aben HP, Luijten L, Jansen BP, Visser-Meily JM, Spikman JM, Biessels GJ (2020). Absence of an infarct on MRI is not uncommon after clinical diagnosis of ischemic stroke. J Stroke Cerebrovasc Dis.

[3] Makin SD, Doubal FN, Dennis MS, Wardlaw JM (2015). Clinically confirmed stroke with negative diffusion-weighted imaging magnetic resonance imaging: longitudinal study of clinical outcomes, stroke recurrence, and systematic review. Stroke.

[4] Kim K, Kim BJ, Huh J, Yang SK, Yang MH, Han MK (2021). Delayed lesions on diffusion-weighted imaging in initially lesion-negative stroke patients. J Stroke.

[5] Mocco J, Siddiqui AH, Fiorella D, Alexander MJ, Arthur AS, Baxter BW (2022). Perfusion imaging selection of ischemic stroke patients for endovascular therapy. J Neurointerv Surg.

[6] Campbell BC, Mitchell PJ, Kleinig TJ, Dewey HM, Churilov L, Yassi N (2015). Endovascular therapy for ischemic stroke with perfusion-imaging selection. N Engl J Med.

[7] Albers GW, Marks MP, Kemp S, Christensen S, Tsai JP, Ortega-Gutierrez S (2018). Thrombectomy for Stroke at 6 to 16 hours with selection by Perfusion Imaging. N Engl J Med.

[8] Biesbroek JM, Niesten JM, Dankbaar JW, Biessels GJ, Velthuis BK, Reitsma JB (2013). Diagnostic accuracy of CT perfusion imaging for detecting acute ischemic stroke: a systematic review and meta-analysis. Cerebrovasc Dis.

[9] Kim Y, Lee S, Abdelkhaleq R, Lopez-Rivera V, Navi B, Kamel H (2021). Utilization and availability of Advanced Imaging in patients with Acute ischemic stroke. Circ Cardiovasc Qual Outcomes.

[10] Stroke, –, 1989 (1989). Recommendations on stroke prevention, diagnosis, and therapy. Report of the WHO Task Force on Stroke and other Cerebrovascular Disorders. Stroke.

[11] Adams HP, Bendixen BH, Kappelle LJ, Biller J, Love BB, Gordon DL (1993). Classification of subtype of acute ischemic stroke. Definitions for use in a multicenter clinical trial. TOAST. Trial of Org 10172 in Acute Stroke Treatment. Stroke.

[12] Lyden P, Brott T, Tilley B, Welch KM, Mascha EJ, Levine S (1994). Improved reliability of the NIH stroke scale using video training. NINDS TPA Stroke Study Group. Stroke.

[13] van Swieten JC, Koudstaal PJ, Visser MC, Schouten HJ, van Gijn J (1988). Interobserver agreement for the assessment of handicap in stroke patients. Stroke.

[14] Zhu C, Qin W, Hu W (2023). Perfusion deficits in different mechanisms of two subtypes of Acute Stroke with Diffusion MRI confirmation. Curr Neurovasc Res.

[15] Shi Z, Li J, Zhao M, Zhang M, Wang T, Chen L (2021). Baseline cerebral ischemic core quantified by different Automatic Software and its predictive value for clinical outcome. Front Neurosci.

[16] Staals J, Makin SD, Doubal FN, Dennis MS, Wardlaw JM (2014). Stroke subtype, vascular risk factors, and total MRI brain small-vessel disease burden. Neurology.

[17] Demeestere J, Wouters A, Christensen S, Lemmens R, Lansberg MG (2020). Review of Perfusion Imaging in Acute ischemic stroke: from time to tissue. Stroke.

[18] Nagaraja N (2021). Diffusion weighted imaging in acute ischemic stroke: a review of its interpretation pitfalls and advanced diffusion imaging application. J Neurol Sci.

[19] Václavík D, Volný O, Cimflová P, Švub K, Dvorníková K, Bar M (2022). The importance of CT perfusion for diagnosis and treatment of ischemic stroke in anterior circulation. J Integr Neurosci.

[20] Estrada UML, Meeks G, Salazar-Marioni S, Scalzo F, Farooqui M, Vivanco-Suarez J (2022). Quantification of infarct core signal using CT imaging in acute ischemic stroke. Neuroimage Clin.

[21] Yu Y, Han Q, Ding X, Chen Q, Ye K, Zhang S (2016). Defining core and Penumbra in ischemic stroke: a Voxel- and volume-based analysis of whole brain CT perfusion. Sci Rep.

[22] Thierfelder KM, Sommer WH, Baumann AB, Klotz E, Meinel FG, Strobl FF (2013). Whole-brain CT perfusion: reliability and reproducibility of volumetric perfusion deficit assessment in patients with acute ischemic stroke. Neuroradiology.

[23] Freeman JW, Luby M, Merino JG, Latour LL, Auh S, Song SS (2013). Negative diffusion-weighted imaging after intravenous tissue-type plasminogen activator is rare and unlikely to indicate averted infarction. Stroke.

[24] Li G, Feng X, Wang C, Hao Y, Wang S, Xiong Y, et al. In-hospital clinical outcomes in diffusion weighted imaging-negative stroke treated with intravenous thrombolysis. BMC Neurol. 2022;22(1:349). 10.1186/s12883-022-02878-w. 10.1186/s12883-022-02878-w PMC9476428 36109692

[25] Ohira J, Ohara N, Hinoda T, Morimoto T, Kohara N (2021). Patient characteristics with negative diffusion-weighted imaging findings in acute lateral medullary infarction. Neurol Sci.

[26] Wang Y, Jing J, Pan Y, Wang M, Meng X, Wang Y (2022). Distribution and prognosis of acute ischaemic stroke with negative diffusion-weighted imaging. Stroke Vasc Neurol.

[27] Hofmeijer J, van Putten MJ (2012). Ischemic cerebral damage: an appraisal of synaptic failure. Stroke.

[28] Kruetzelmann A, Köhrmann M, Sobesky J, Cheng B, Rosenkranz M, Röther J (2011). Pretreatment diffusion-weighted imaging lesion volume predicts favorable outcome after intravenous thrombolysis with tissue-type plasminogen activator in acute ischemic stroke. Stroke.

[29] Seyhan M, Mackenrodt D, Gunreben I, Müllges W, Haeusler KG, Pham M (2020). Should IV Thrombolysis be given in patients with suspected ischemic stroke but unknown Symptom Onset and without diffusion-weighted imaging lesion? - results of a case-control study. J Stroke Cerebrovasc Dis.

